# Exploring the role of Sichuan Baoning vinegar microbiota and the association with volatile flavor compounds at different fermentation depths

**DOI:** 10.3389/fmicb.2023.1135912

**Published:** 2023-02-15

**Authors:** Aiping Liu, Yixue Ou, Haojie Shu, Tianyu Mou, Qin Li, Jianlong Li, Kaidi Hu, Shujuan Chen, Li He, Jiang Zhou, Xiaolin Ao, Yong Yang, Shuliang Liu

**Affiliations:** ^1^College of Food Science, Sichuan Agricultural University, Ya’an, Sichuan, China; ^2^Sichuan Baoning Vinegar Co., Ltd., Langzhong, Sichuan, China

**Keywords:** cereal vinegar, high-throughput sequencing, microbiota, Sichuan Baoning vinegar, volatile flavor compounds

## Abstract

Cereal vinegar is usually produced through solid-state fermentation, and the microbial community plays an important role in fermentation. In this study, the composition and function of Sichuan Baoning vinegar microbiota at different fermentation depths were evaluated by high-throughput sequencing combined with PICRUSt and FUNGuild analysis, and variations in volatile flavor compounds were also determined. The results revealed that no significant differences (*p* > 0.05) were found in both total acid content and pH of vinegar *Pei* collected on the same day with different depths. There were significant differences between the bacterial community of samples from the same day with different depths at both phylum and genus levels (*p* < 0.05), however, no obvious difference (*p* > 0.05) was observed in the fungal community. PICRUSt analysis indicated that fermentation depth affected the function of microbiota, meanwhile, FUNGuild analysis showed that there were variations in the abundance of trophic mode. Additionally, differences in volatile flavor compounds were observed in samples from the same day with different depths, and significant correlations between microbial community and volatile flavor compounds were observed. The present study provides insights into the composition and function of microbiota at different depths in cereal vinegar fermentation and quality control of vinegar products.

## 1. Introduction

Vinegar is a popular traditional acidic condiment and preservative with a long history (Li et al., 2015). Production of vinegar is usually divided into liquid-state fermentation and solid-state fermentation according to the fermentation type (Haruta et al., 2006; Li et al., 2022). In Europe, vinegar is mainly produced by liquid-state fermentation using fruits as raw materials; in Asia such as China, Japan, and Korea vinegar is mainly produced by solid-state fermentation using cereals as raw materials (Lynch et al., 2019; Yuan et al., 2021). Compared to liquid-state fermentation, solid-state fermentation involves more complex microbiota, leading to more abundant flavor (Wu et al., 2019; Gao et al., 2022). However, in order to enhance production yield and efficiency, two-stage liquid–solid fermentation is also utilized by Chinese vinegar manufacturers (Liu et al., 2022). Sichuan Baoning vinegar, as representative of Sichuan bran vinegar, is one of the four traditionally famous vinegars in China (Chen et al., 2016). Sichuan Baoning vinegar differs from other vinegars in the following aspects: use of uncooked wheat bran as the primary raw material and Daqu (great koji) incorporated with Chinese herbs as the saccharifying agent; complete solid-state fermentation; an open fermentation process, with saccharification, alcohol fermentation, and acetic acid fermentation carried out simultaneously. The lactic acid content in Baoning vinegar was higher, leading to a more soft taste (Liu et al., 2022).

As is known, the processing of vinegar relies on a variety of microorganisms, and both culture-dependent (Wu et al., 2010; Vegas et al., 2013) and culture-independent (Kong et al., 2022; Sengun et al., 2022) methods have been employed to study the microbiota during vinegar fermentation. Due to the open fermentation of cereal vinegar, the microbiota succession may be affected by environmental factors and microorganisms from raw materials, processing equipment, and surrounding environment. The contents of ethanol, titratable acid, and reducing sugar, rather than temperature, were reported to be the major environmental factors shaping the microbiota of Zhenjiang aromatic vinegar (Huang et al., 2022). Meanwhile, fermentation depth may also impose an effect on solid-state fermentation since the depth of the substrate can affect the magnitude of temperature and oxygen gradients (Rathbun and Shuler, 1983; Ridder et al., 1999). The volatile flavor compounds are reliable quality indicators of vinegar. The relationship between volatile flavor compounds and microbial community has been confirmed (Wang et al., 2022; Zhang et al., 2022). However, little is known about the effects of fermentation depth on the microbiota and volatile flavor compounds of cereal vinegar.

In the present study, amplicon-based high-throughput sequencing was applied to explore the composition and function of Sichuan Baoning vinegar microbiota at the upper and lower layers. Meanwhile, the relationship between microbiota and volatile flavor compounds was studied, expecting to provide insights into the fermentation mechanism and quality control of cereal vinegar.

## 2. Materials and methods

### 2.1. Sampling

The vinegar *Pei* samples (the fermentation mixture before leaching) were collected from Sichuan Baoning Vinegar Co., Ltd. (Langzhong, Sichuan, China) between July 2022 and August 2022. The production of Baoning vinegar was carried out as reported by Liu et al. (2021), and samples (obtained approximately 0.5 ~ 1 h before mixing, except for the 1st day’s sample obtained ~ 1 h after mixing) on days 1, 5, 9, 13, 17, 21, 24, and 27 from the upper (5 ~ 40 cm from the top) and lower (70 ~ 110 cm from the top) layers were collected using the five-point sampling method (four corners and center of each layer), respectively. Samples from the same layer were mixed, and excess vinegar *Pei* was removed by the quadrate method. Afterward, 300 ~ 400 g of vinegar *Pei* was sealed in sterile Ziplock bags at –40°C. Samples are identified based on the sampling day and the layer (U = upper, L = lower), e.g., U1 indicates upper layer samples collected on day 1. The total acid content of vinegar *Pei* was recorded as reported by Liu et al. (2020), and pH was determined using a pH meter (PHS–3CB; Shanghai Yueping Scientific Instrument Co., Ltd., Shanghai, China).

### 2.2. Miseq sequencing

Each sample was divided into three portions and sent to Majorbio (Shanghai, China) for bacterial and fungal diversity analysis using the Illumina Miseq system, e.g., sample U1 was sequenced with three portions identified as U1_1, U1_2, and U1_3. Specifically, primers 338F (5′-ACTCCTACGGGAGGCAGCAG′) and 806R (5′-GGACTACHVGGGTWTCTAAT-3′) were used to amplify the V3–V4 regions of bacterial 16S rDNA, while fungal internal transcribed spacer (ITS) regions were amplified using primers ITS1F (5′-CTTGGTCATTTAGAGGAAGTAA-3′) and ITS2R (5′-GCTGCGTTCTTCATCGATGC-3′; Zeng et al., 2022).

### 2.3. Bioinformatics analysis

After sequencing, PE reads were spliced according to the overlap relationship using Flash (version 1.2.11), and then trimming, quality control, and assessment of raw reads were conducted by fastp (version 0.19.6). Similar sequences were clustered into the same operational taxonomy unit (OTU) with a 97% sequence identity. The OTU number of each sample was employed to assess species richness. The sequences were evaluated for alpha-diversity (Mothur, version 1.30.2) and beta-diversity (Qiime, version 1.9.1). Linear discriminant analysis (LDA) effect size (LEfSe) was used to analyze the different species at different fermentation depths using LEfSe software (version 1.0), and an LDA score ≥ 2.0 was set to indicate important biomarkers in samples. Microbial functions were annotated by PICRUSt2 (version 2.2.0) for the 16S rDNA OTU and FUNGuild (version 1.0) for the ITS OTU.

### 2.4. Volatile flavor compounds analysis

The volatile flavor compounds were tested as previously reported (Liu et al., 2020) using gas chromatography–mass spectrometry (GC–MS; 5975C/6890 N; Agilent, United States) with an elastic capillary vessel column (HP-5MS, 30 m × 0.25 mm i.d., 0.25 μm film thickness). After comparing with the NIST 2020 library, compounds with a similarity index higher than 80% were employed for analysis. After normalization of all peak areas, the relative content of each compound was calculated based on its peak area compared to all peak areas.

### 2.5. Statistical analysis

The total acid, pH, and volatile flavor compounds of vinegar *Pei* samples were analyzed with three independent replicates and expressed as mean ± SD. The relationship between total acid, pH, and microbial community structure was analyzed by RDA/CCA. Spearman’s rank correlation coefficient was used to analyze the relationship between volatile flavor compounds and microbiota. Statistical comparative analysis between two groups was performed by t-test using SPSS 27.0 (SPSS, Chicago, IL, United States) unless otherwise specified.

## 3. Results and discussion

### 3.1. Microbiota analysis

#### 3.1.1. Alpha diversity

Alpha diversity indices, including shannon, chao, and coverage, were calculated in different samples (Supplementary Table S1). The coverage of sequences was over 0.999, indicating sufficient authenticity and depth of sequencing (Zhao et al., 2022). The shannon and chao values in the bacterial and fungal communities were similar between most of vinegar *Pei* from the same day with different fermentation depths. As revealed by shannon indice, the bacterial community diversity of the upper layer was lower than that of the lower layer on day 13 (*p* < 0.05), but it was higher between day 17 to 24 (*p* > 0.05), especially on day 27 (*p* < 0.05). As revealed by chao value, the bacterial richness exhibited an opposite trend after day 17, and significant differences were observed on days 24 and 27. For the fungal community, the richness of the upper layer was significantly lower than that of the lower layer on day 17 (*p* < 0.05), but it became higher after day 24, especially on day 27 (*p* < 0.05). The diversity of the fungal community in both layers was similar, except for day 5.

#### 3.1.2. Beta diversity

**Figure 1:**
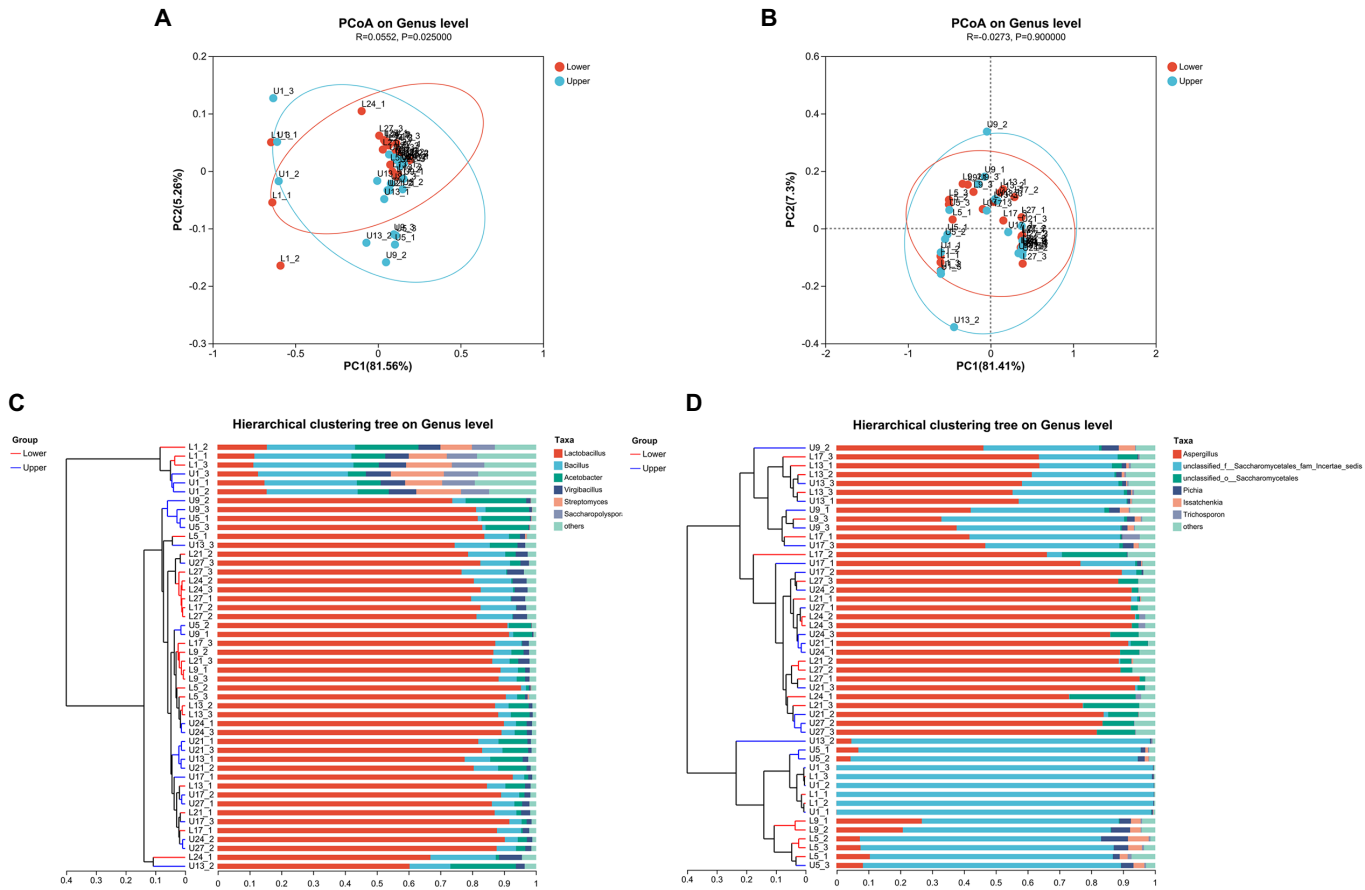
Principal coordinates analysis (PCoA) **(A)** and unweighted pair-group method with arithmetic mean (UPGMA) **(C)** analysis of bacterial community structure; PCoA **(B)** and UPGMA **(D)** analysis of fungal community structure.

The principal coordinates analysis (PCoA) at genus level based on the Bray-Curtis distance indicated that the first two principal components make up 81.56 and 5.26% of the total variance contribution ratio in the bacterial community (Figure 1A), whereas the first two principal components make up 81.41 and 7.30% of the total variance contribution ratio in the fungal community (Figure 1B). There were significant differences in the bacterial community of samples from different depths at genus level (*p* < 0.05), but few differences were observed in the fungal community (*p* > 0.05). The unweighted pair-group method with arithmetic mean (UPGMA) distance analysis on genus level revealed that most of the samples from the same day with different depths were separated (Figures 1C,D).

#### 3.1.3. Bacterial and fungal communities

**Figure 2:**
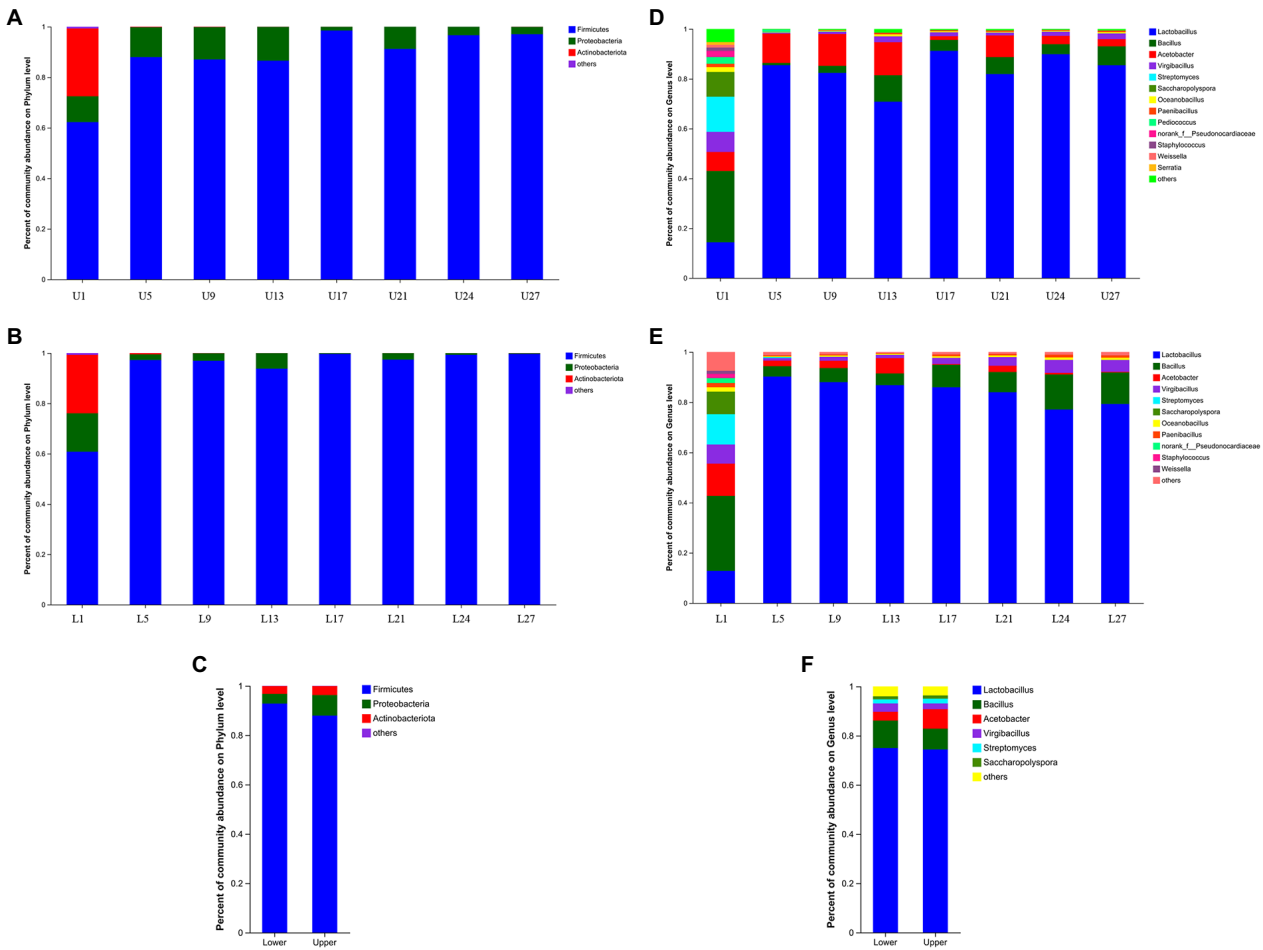
Relative abundance of the bacterial community structure. **(A)** The upper layer at phylum level, **(B)** the lower layer at phylum level, **(C)** the upper and lower layers at phylum level, **(D)** the upper layer at genus level, **(E)** the lower layer at genus level, **(F)** the upper and lower layers at genus level.

A total of 194 OTUs were obtained based on 97% similarity threshold, and 7 fungal phyla, 13 fungal classes, 38 fungal orders, 58 fungal families, and 94 fungal genera were detected in vinegar *Pei* samples from both fermentation layers. At the phylum level, Firmicutes, Proteobacteria, and Actinobacteriota all possessed the highest abundance through the fermentation in both fermentation depths, which was similar to our previous report, but the abundance of Cyanobacteria, Bacteroidetes, and Deinococcus-Thermus was lower in the present study (Liu et al. (2021)). The abundance of Proteobacteria and Actinobacteriota was a little higher in the upper layer while that of Firmicutes was higher in the lower layer (Figures 2A–C). At the genus level, similar to our previous report (Liu et al., 2022), *Lactobacillus*, *Bacillus*, and *Acetobacter* were the top 3 genera through the fermentation in both fermentation depths (Figures 2D,E), but the abundance of *Lactobacillus* (74.98%) and *Bacillus* (11.14%) was higher in the lower layer while the abundance of *Acetobacter* (7.94%) was higher in the upper layer (Figure 2F). On the first day of fermentation, *Bacillus* was the predominant (28.64%), followed by *Lactobacillus* (14.35%) and *Streptomyces* (14.11%), which was different from previous reports (Liu et al. (2021); Liu et al., 2022) and the reason might lie in the differences in environmental factors and microorganisms from materials, processing equipment, and surrounding environment. Afterward, *Lactobacillus* was predominant, ranging from 70.83 to 91.23% in the upper layer and 77.13 to 90.24% in the lower layer, respectively, which was not in agreement with the results of Zhenjiang aromatic vinegar and Shanxi mature vinegar (Huang et al., 2022; Kou et al., 2022). Generally, the abundance of *Lactobacillus* showed an increasing and then decreasing trend during the fermentation in the lower layer whereas no regular pattern was observed in the upper layer. The abundance of *Acetobacter* also changed without a regular pattern, but it was the highest on day 13 in both fermentation depths.

**Figure 3:**
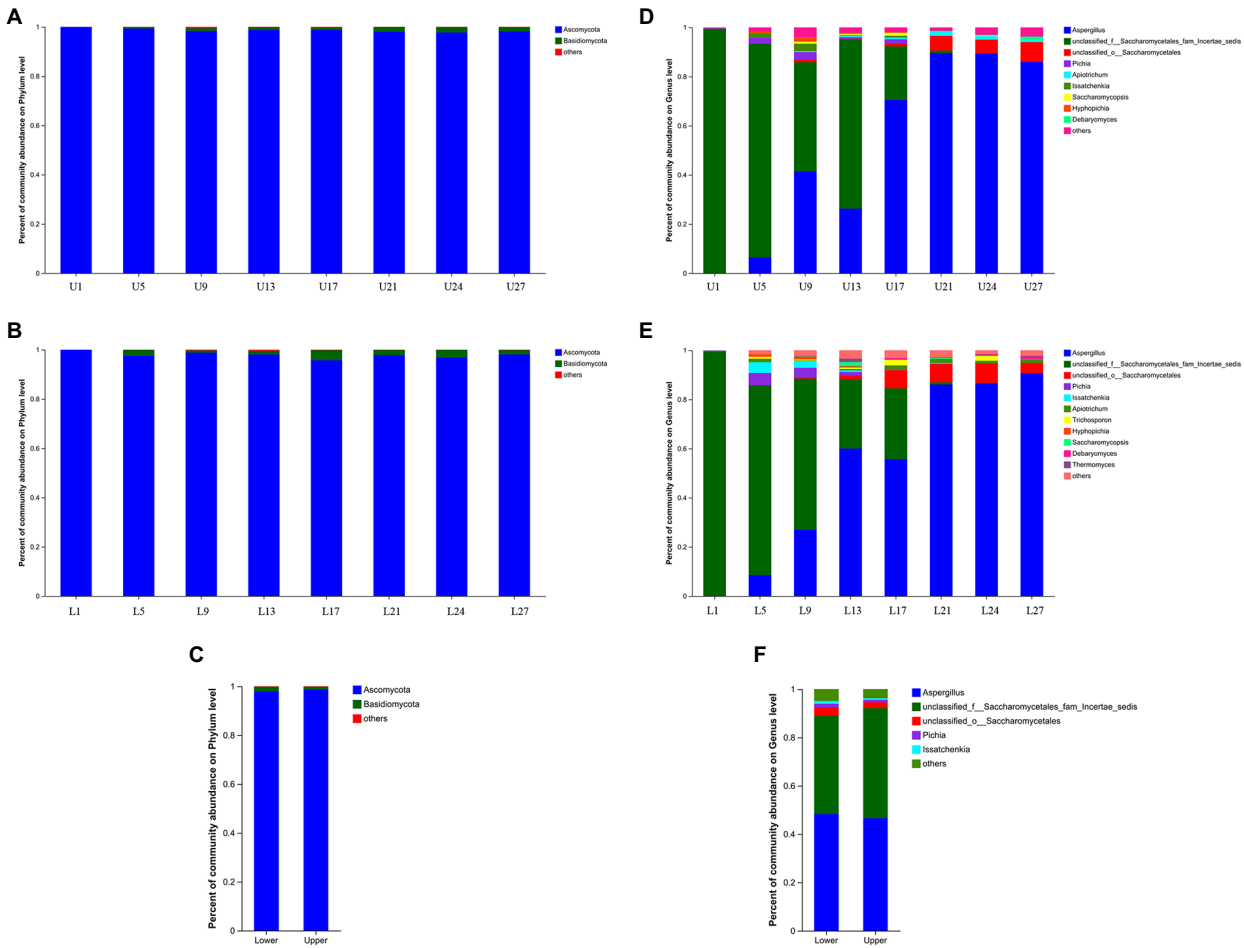
Relative abundance of the fungal community structure. **(A)** The upper layer at phylum level, **(B)** the lower layer at phylum level, **(C)** the upper and lower layers at phylum level, **(D)** the upper layer at genus level, **(E)** the lower layer at genus level, **(F)** the upper and lower layers at genus level.

A total of 649 OTUs were obtained based on 97% similarity threshold, and 9 fungal phyla, 30 fungal classes, 69 fungal orders, 166 fungal families, and 316 fungal genera were detected in vinegar *Pei* samples from both fermentation layers. At the phylum level, Ascomycota and Basidiomycota possessed the highest abundance through fermentation in both fermentation depths, and the abundance of Ascomycota was far higher than that of Basidiomycota, which agreed with the study by Ai et al. (2019). Notably, the abundance of Ascomycota was higher in the lower layer (Figures 3A–C). At the genus level, *Aspergillus* was the predominant, followed by unclassified_f__Saccharomycetales_fam_Incertae_sedis, unclassified_o__Saccharomycetales, *Pichia* and *Apiotrichum* in the upper layer, while the predominant genera in the lower layer were *Aspergillus*, unclassified_f__Saccharomycetales_fam_Incertae_sedis, unclassified_o__Saccharomycetales, *Pichia* and *Issatchenkia* (Figures 3D,E). Generally, the abundance of *Aspergillus* increased with the fermentation, while unclassified_f__Saccharomycetales_fam_Incertae_sedis showed a decreasing trend. The changing trend of unclassified_o__Saccharomycetales, *Pichia*, and *Issatchenkia* were different in the upper and lower layers. The primary difference in the upper and lower layers was that the abundance of *Aspergillus* (48.30%), *Pichia* (1.49%), and *Issatchenkia* (1.11%) was higher at the lower layer (Figure 3F), which might be caused by more accessible oxygen in the upper layer.

**Figure 4:**
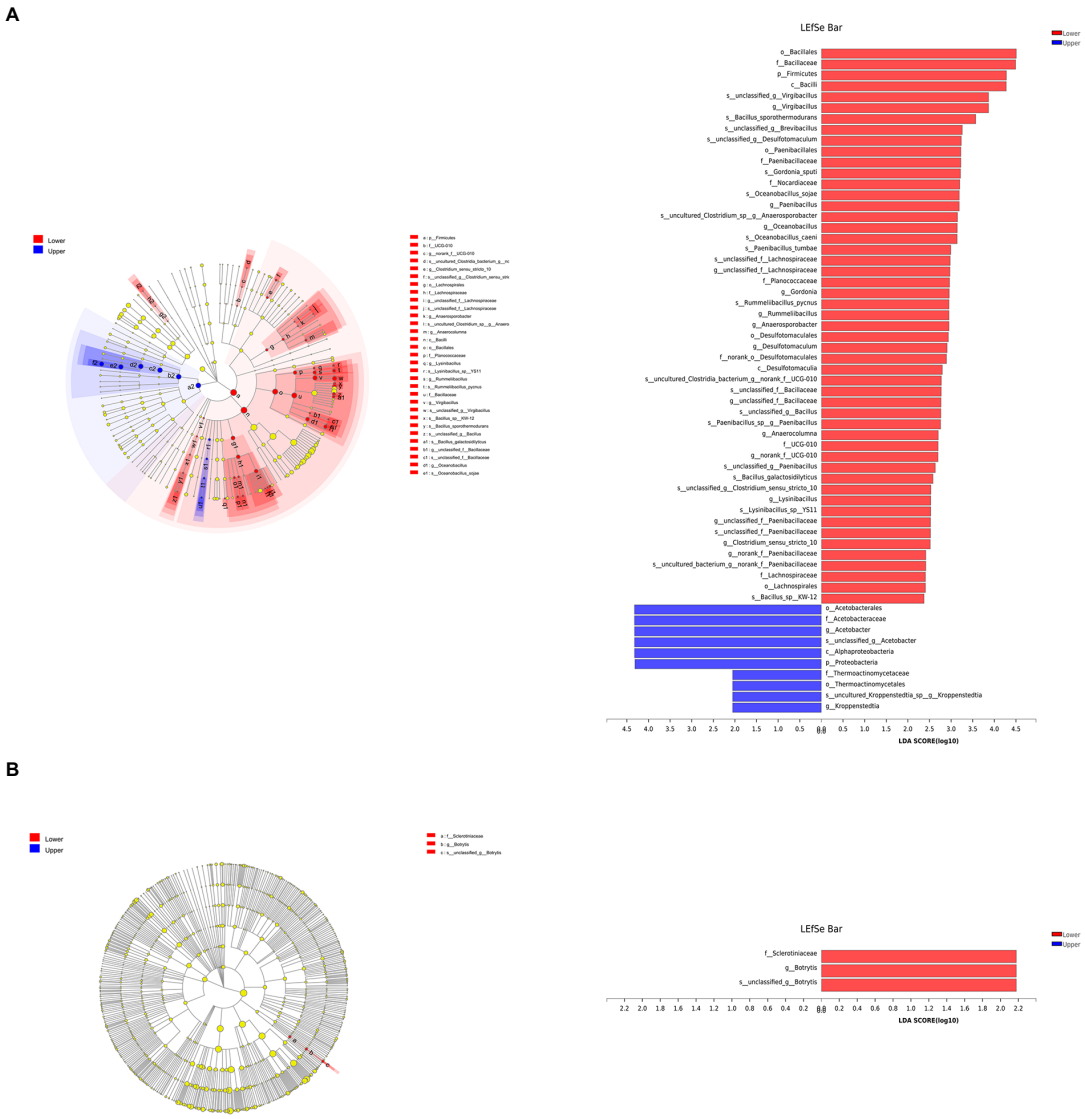
The bacterial **(A)** and fungal **(B)** communities of vinegar *Pei* were analyzed through Linear discriminant analysis effect size (LEfSe) to determine the optimal characteristic taxa.

Linear discriminant analysis effect size analysis (logarithmic LDA scores threshold of 2) was performed to identify bacterial (Figure 4A) and fungal (Figure 4B) taxa with significant abundance differences in different layers. *Virgibacillus*, *Paenibacillus*, *Oceanobacillus*, and 12 other bacterial genera were significantly enriched in the lower layer, while bacterial genera *Acetobacter* and *Kroppenstedtia* were significantly enriched in the upper layer. Fungal genus *Botrytis* was significantly enriched in the lower layer.

Analysis of similarities (ANOSIM; Supplementary Table S2) indicated that no significant differences were observed in the bacterial community of samples from the same day with different fermentation depths at OTU level (*p* > 0.05), however, there were significant differences between the bacterial community of samples from the same day with different fermentation depths at both phylum and genus levels (*p* < 0.05). As for the fungal community, no significant differences were observed in samples from different fermentation depths at OTU, phylum, and genus levels (*p* > 0.05).

#### 3.1.4. PICRUSt and FUNGuild functional prediction

**Figure 5:**
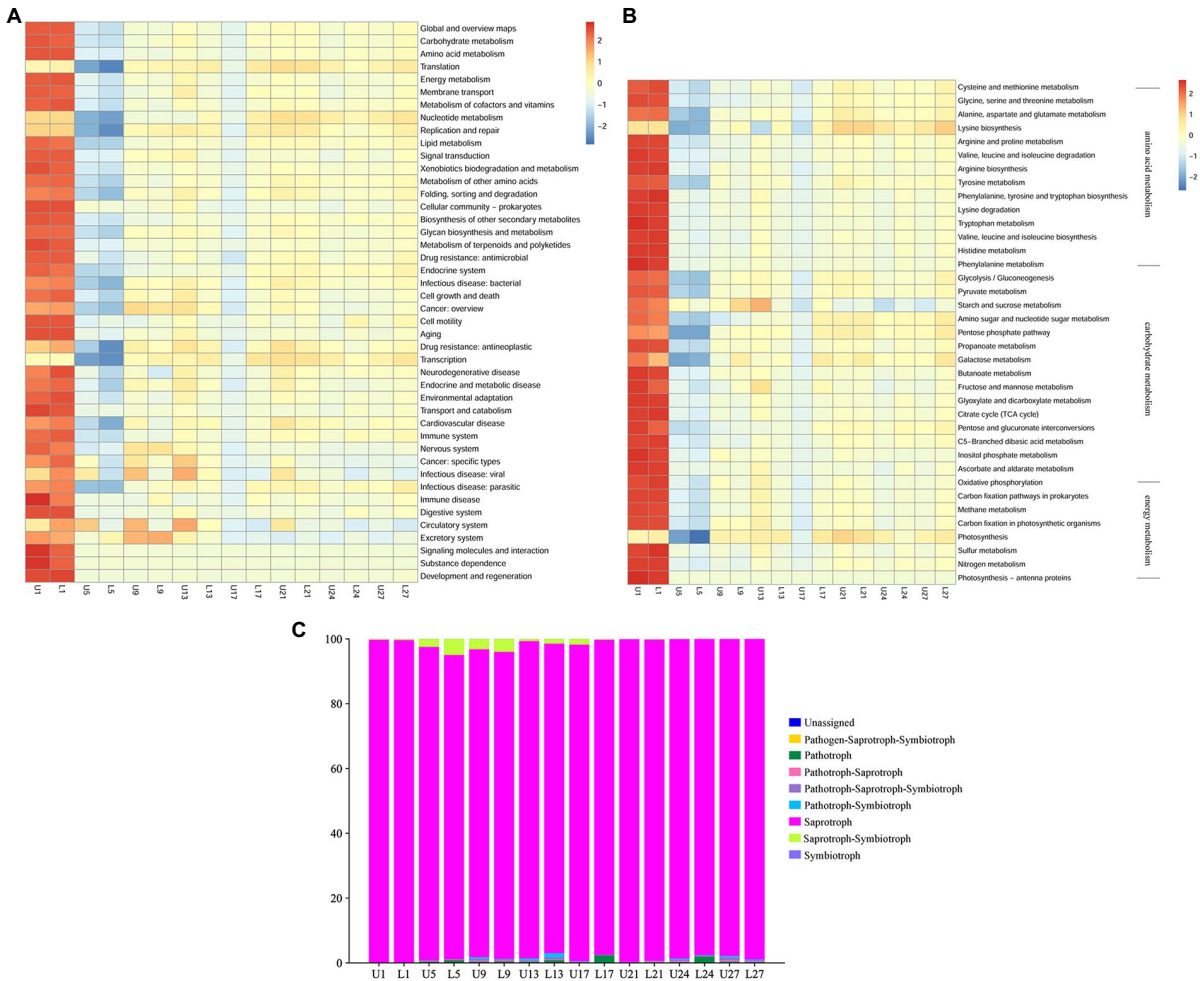
Heatmap showing the hierarchical clustering of predicted KEGG Orthologs functional profiles of the bacterial community at level 2 **(A)** and level 3 (carbohydrate, amino acid, and energy metabolism) **(B)**; Relative abundance of predicted trophic mode of fungi **(C)**.

PICRUSt2 was applied to predict the bacterial function, and 6 types of biological metabolic pathways, including metabolism, genetic information processing, environmental information processing, human diseases, cellular processes, and organismal systems, were obtained by comparing with the KEGG database. Forty-four subfunctions were observed in the analysis of the secondary functional layer of the predicted genes, and differences were found between samples on the same day with different fermentation depths, especially for cell motility, circulatory system, and excretory system, etc. (Figure 5A). Metabolism was the primary pathway, and carbohydrate, amino acid, and energy metabolism of the microbiota were predicted (Figure 5B). As can be seen, more variations were observed at the third level. As for carbohydrate metabolism, the most obvious difference was in starch and sucrose metabolism.

FUNGuild was used to predict the function of the fungal community. As illustrated in Figure 5C, eight trophic modes were classified, and the “unassigned” was defined as OTUs that were not matched with any taxa. Saprotroph was the primary trophic mode throughout the fermentation, with relative abundance ranging from 93.88 to 99.72%. Saprotroph-symbiotroph was the second most abundant trophic mode in samples from day 1 to 13, while the type of the second most abundant trophic mode varied after day 17.

### 3.2. Content of total acid and pH of vinegar *Pei*

**Figure 6:**
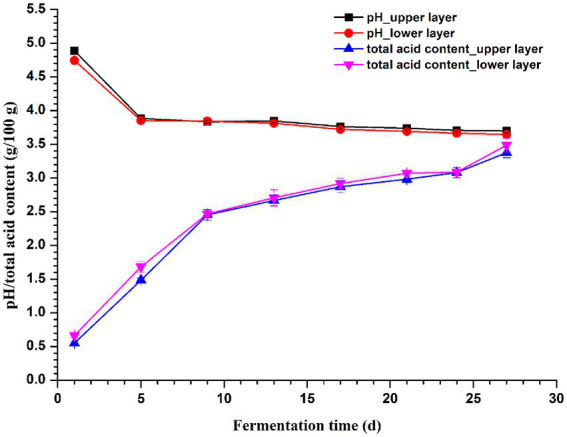
Changes in pH and total acid content of vinegar *Pei* during fermentation.

As shown in Figure 6, the total acid content increased with the fermentation in both layers, while pH showed a decreasing trend. Notably, there was no significant difference (*p* > 0.05) in both total acid content and pH of vinegar *Pei* collected on the same day with different depths, which might indicate a good processing control on vinegar production to some extent. RDA/CCA revealed that total acid and pH significantly affected the microbial community, and these two factors together explained 84.64 and 60.39% of the bacterial and fungal communities variation at genus level, respectively (Supplementary Figures S1A,C). The correlation between pH/total acid content and the top 10 abundant genera was evaluated by the spearman correlation heatmap. Both pH and total acid content were significantly correlated with bacterial genera *Acetobacter*, *Streptomyces*, *Saccharopolyspora*, and *Pediococcus* (Supplementary Figure S1B), while significant correlations were observed between pH/total acid content and all the 10 most abundant fungal genera except *Trichisporon* and *Saccharomycopsis* (Supplementary Figure S1D; *p* < 0.001).

### 3.3. Volatile flavor compounds

The number of six common types of volatile flavor compounds, including esters, acids, alcohols, carbonyls, heterocyclic, and olefines, as well as their relative percentage composition were shown in Table 1. There were certain differences in both the number of volatile flavor compounds and their relative percentage composition between samples from the same day with different depths. Obvious differences were observed in the relative contents of acids and alcohols, which were primarily introduced by the content of acetic acid and phenethyl alcohol, respectively. Both the type and percentage of ester compounds were the most abundant throughout fermentation, with the relative percentage composition ranging from 26.97 to 50.25% after day 1, which agreed with the result by Jiang et al. (2019). Similar to the report by Gong et al. (2021), the relative content of alcohols was the second highest. After the beginning of fermentation, obvious differences in the relative percentage composition of ester compounds and acid compounds were observed on day 13, whereas that of alcohol compounds and carbonyl compounds were observed on days 9 and 9–13, respectively. Ethyl octanoate, ethyl acetate, ethyl caproate, phenethyl acetate, and ethyl phenylacetate, detected in various vinegars (Ríos-Reina et al., 2020; Pothimon et al., 2022; Xu et al., 2022), were esters with high relative percentage. Tetramethyl pyrazine, a characteristic flavor and bioactive compound in vinegar (Li et al., 2016; Liu et al., 2021), was detected in vinegar *Pei* from both depths, and there was no significant difference in its relative content (*p* > 0.05). Notably, phenols such as 2-methoxy-5-methylphenol and 4-ethyl-2-methoxy-phenol, rarely reported in cereal vinegar, were produced, and 4-ethyl-2-methoxy-phenol was detected throughout the fermentation. Observable differences in their relative percentage were found between samples from the same day with different depths (data not shown).

**Table 1:** Number of volatile flavor compounds and their relative percentage composition (n/%) during vinegar fermentation.

Sample	Esters		Acids		Alcohols		Carbonyls		Heterocyclic		Olefines	
	Type	Percentage	Type	Percentage	Type	Percentage	Type	Percentage	Type	Percentage	Type	Percentage
U1	13	15.77 ± 0.76	6	35.31 ± 3.83	4	4.11 ± 0.68	10	4.19 ± 0.43	5	7.34 ± 1.04	1	3.88 ± 1.08
L1	17	14.05 ± 4.38	5	14.78 ± 4.96	7	23.31 ± 4.54	9	6.64 ± 1.09	4	8.87 ± 1.58	1	3.70 ± 0.18
U5	29	50.25 ± 4.65	5	1.05 ± 0.40	10	18.84 ± 3.55	7	5.66 ± 1.48	3	3.34 ± 1.39	1	0.42 ± 0.36
L5	22	48.33 ± 0.81	7	5.09 ± 1.50	10	16.60 ± 2.40	5	3.65 ± 0.71	2	7.52 ± 0.38	1	0.42 ± 0.42
U9	29	45.84 ± 3.64	7	13.84 ± 0.36	14	13.63 ± 4.00	7	5.41 ± 1.27	3	5.82 ± 1.54	3	0.81 ± 0.80
L9	27	35.81 ± 4.56	6	13.03 ± 5.18	15	27.20 ± 2.37	2	2.54 ± 0.69	3	3.71 ± 0.56	0	–
U13	21	26.97 ± 1.27	7	22.28 ± 5.27	15	20.34 ± 194	7	8.22 ± 0.43	4	5.30 ± 2.82	0	–
L13	25	45.48 ± 3.62	6	6.71 ± 0.74	11	19.85 ± 3.78	5	2.77 ± 0.48	2	6.24 ± 1.17	0	–
U17	25	31.23 ± 2.77	6	16.31 ± 7.39	13	20.94 ± 2.53	4	3.48 ± 0.68	3	4.28 ± 0.57	0	–
L17	21	28.89 ± 1.34	7	12.72 ± 3.21	13	18.09 ± 1.16	4	3.16 ± 0.92	3	4.29 ± 0.27	0	–
U21	24	36.45 ± 4.23	8	23.06 ± 1.69	17	23.42 ± 2.07	9	5.88 ± 3.01	1	0.68 ± 0.13	0	–
L21	24	32.60 ± 2.19	7	12.32 ± 0.84	14	22.28 ± 6.92	4	3.35 ± 1.14	3	3.73 ± 0.01	0	–
U24	24	40.60 ± 2.81	5	12.58 ± 7.44	13	22.13 ± 3.58	5	4.10 ± 0.40	2	4.08 ± 1.05	0	–
L24	23	35.83 ± 10.54	7	12.37 ± 2.73	13	28.35 ± 9.06	2	2.53 ± 0.05	2	3.02 ± 0.63	0	–
U27	26	42.00 ± 1.71	7	10.39 ± 4.89	14	23.87 ± 5.23	5	4.12 ± 0.70	3	2.38 ± 0.71	0	–
L27	27	39.79 ± 3.12	7	7.80 ± 1.50	15	27.90 ± 4.14	8	3.34 ± 0.58	2	1.42 ± 0.77	1	0.28 ± 0.24

-, not detected.

**Figure 7:**
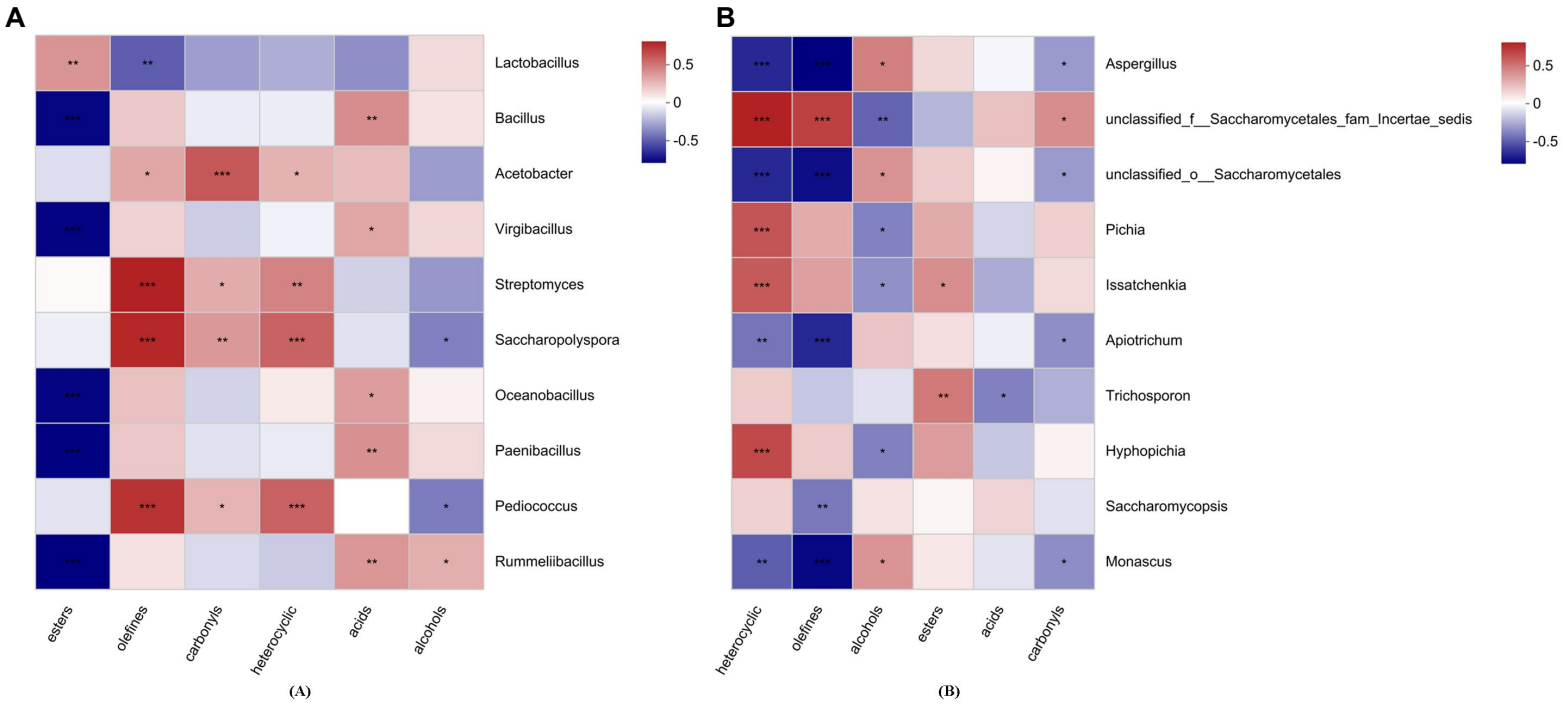
The correlation between volatile flavor compounds and the top 10 bacterial **(A)** and fungal **(B)** genera. ^*^p < 0.05, ^**^p < 0.01, ^***^p < 0.001.

The correlation between volatile flavor compounds and the 10 most abundant genera was revealed by the spearman correlation heatmap (Figure 7). Esters and acids showed significant correlations with *Bacillus*, *Virgibacillus*, *Oceanobacillus*, *Paenibacillus*, *Rummeliibacillus*, and *Trichosporon*. *Acetobacter* was significantly positively correlated with olefins, carbonyls and heterocyclic compounds (*p* < 0.05). Alcohols showed a significant correlation with *Pediococcus*, *Aspergillus*, *unclassified_f__Saccharomycetales_fam_Incertae_sedis*, *unclassified_o__Saccharomycetales*, *Pichia, Saccharopolyspora, Rummeliibacillus, Issatchenkia, Monascus*, and *Hyphopichia* (*p* < 0.05).

## 4. Conclusion

Significant differences between the bacterial community of samples from the same day with different fermentation depths at both phylum and genus levels were observed (*p* < 0.05), whereas no significant difference was observed in the fungal community. *Acetobacter* and *Kroppenstedtia* were significantly enriched in the upper layer. The function of microbiota was altered according to PICRUSt analysis, and the number of volatile flavor compounds and their relative percentage composition were affected by the microbial community at different depths. However, no significant difference was observed in total acid content. To the best of our knowledge, this has been the first study trying to explore the effects of fermentation depth on vinegar fermentation, which will provide insights into the quality control of cereal vinegar production. The changes in other vinegar quality attributes and detailed mechanisms under the effects of fermentation depth on microbiota composition and function need further study.

## Data availability statement

The raw data supporting the conclusions of this article will be made available by the authors, without undue reservation.

## Author contributions

AL: conceptualization, writing—original draft, writing—review and editing, and funding acquisition. YO, HS, and TM: methodology and data curation. QL, JL, KH, SC, and LH: writing—original draft. JZ: suggestions and resources. XA and YY: writing—review and editing. SL: writing—review and editing and supervision. All authors contributed to the article and approved the submitted version.

## Funding

We appreciate the financial support from the Science and Technology Department of Sichuan Province (No. 2022NSFSC0116 and No. 2020YFN0133).

## Conflict of interest

JZ is employed by Sichuan Baoning Vinegar Co., Ltd.

The remaining authors declare that the research was conducted in the absence of any commercial or financial relationships that could be construed as a potential conflict of interest.

## Publisher’s note

All claims expressed in this article are solely those of the authors and do not necessarily represent those of their affiliated organizations, or those of the publisher, the editors and the reviewers. Any product that may be evaluated in this article, or claim that may be made by its manufacturer, is not guaranteed or endorsed by the publisher.
