# Supplementary material for: Exploring the role of Sichuan Baoning vinegar microbiota and the association with volatile flavor compounds at different fermentation depths
Source: Front Microbiol. 2023 Feb 15;14:1135912. doi: 10.3389/fmicb.2023.1135912 (PMC9975336; doi:10.3389/fmicb.2023.1135912)

Supplementary Material

Exploring the role of Sichuan Baoning vinegar microbiota and the association with volatile flavor compounds at different fermentation depths

Aiping Liu, Yixue Ou, Haojie Shu, Tianyu Mou, Qin Li, Jianlong Li, Kaidi Hu, Shujuan Chen, Li He, Jiang Zhou, Xiaolin Ao, Yong Yang, Shuliang Liu*

*** Correspondence:** Shuliang Liu, lsliang999@163.com

# Supplementary Tables

**Table S1 Estimation of bacterial and fungal communities diversity**

| Sample\Estimators | Bacteria | | | Sample\Estimators | Fungi | | |
| --- | --- | --- | --- | --- | --- | --- | --- |
|  | Shannon | Chao | Coverage |  | Shannon | Chao | Coverage |
| U1_1 | 3.016 | 154.071 | 0.999 | U1_1 | 0.092 | 85.000 | 1.000 |
| U1_2 | 2.831 | 156.938 | 0.999 | U1_2 | 0.028 | 31.600 | 1.000 |
| U1_3 | 2.924 | 168.188 | 0.999 | U1_3 | 0.049 | 56.000 | 1.000 |
| U5_1 | 1.580 | 78.000 | 1.000 | U5_1 | 0.543 | 126.500 | 1.000 |
| U5_2 | 1.337 | 79.143 | 1.000 | U5_2 | 0.515 | 134.353 | 1.000 |
| U5_3 | 1.513 | 106.500 | 0.999 | U5_3 | 0.849 | 127.500 | 1.000 |
| U9_1 | 1.384 | 68.333 | 1.000 | U9_1 | 1.531 | 122.500 | 1.000 |
| U9_2 | 1.695 | 112.500 | 0.999 | U9_2 | 1.530 | 165.000 | 0.999 |
| U9_3 | 1.592 | 92.000 | 0.999 | U9_3 | 1.312 | 148.000 | 1.000 |
| U13_1 | 1.896 | 88.000 | 1.000 | U13_1 | 1.260 | 89.000 | 1.000 |
| U13_2 | 2.094 | 189.333 | 0.999 | U13_2 | 0.313 | 89.545 | 1.000 |
| U13_3 | 2.049 | 126.167 | 0.999 | U13_3 | 1.402 | 134.588 | 1.000 |
| U17_1 | 0.972 | 65.600 | 1.000 | U17_1 | 0.883 | 72.111 | 1.000 |
| U17_2 | 1.134 | 83.111 | 0.999 | U17_2 | 0.577 | 71.600 | 1.000 |
| U17_3 | 0.998 | 91.000 | 0.999 | U17_3 | 1.288 | 89.333 | 1.000 |
| U21_1 | 0.879 | 57.231 | 1.000 | U21_1 | 0.420 | 45.000 | 1.000 |
| U21_2 | 0.926 | 85.600 | 1.000 | U21_2 | 0.725 | 45.000 | 1.000 |
| U21_3 | 0.843 | 75.375 | 0.999 | U21_3 | 0.360 | 42.500 | 1.000 |
| U24_1 | 0.543 | 63.600 | 1.000 | U24_1 | 1.075 | 119.333 | 1.000 |
| U24_2 | 0.534 | 59.500 | 1.000 | U24_2 | 0.993 | 111.167 | 1.000 |
| U24_3 | 0.568 | 53.429 | 1.000 | U24_3 | 1.128 | 125.400 | 1.000 |
| U27_1 | 0.701 | 77.000 | 0.999 | U27_1 | 1.114 | 132.474 | 1.000 |
| U27_2 | 0.626 | 70.750 | 1.000 | U27_2 | 1.329 | 145.526 | 1.000 |
| U27_3 | 0.837 | 65.111 | 1.000 | U27_3 | 1.299 | 124.056 | 1.000 |
| L1_1 | 3.023 | 154.929 | 1.000 | L1_1 | 0.026 | 56.000 | 1.000 |
| L1_2 | 2.835 | 166.056 | 0.999 | L1_2 | 0.038 | 68.000 | 1.000 |
| L1_3 | 2.933 | 162.667 | 0.999 | L1_3 | 0.063 | 53.500 | 1.000 |
| L5_1 | 1.757 | 109.000 | 0.999 | L5_1 | 1.019 | 108.000 | 1.000 |
| L5_2 | 1.240 | 83.000 | 1.000 | L5_2 | 0.939 | 150.091 | 1.000 |
| L5_3 | 1.465 | 112.429 | 0.999 | L5_3 | 0.914 | 157.063 | 1.000 |
| L9_1 | 1.668 | 116.000 | 0.999 | L9_1 | 1.213 | 125.400 | 1.000 |
| L9_2 | 1.737 | 120.333 | 0.999 | L9_2 | 1.245 | 163.000 | 1.000 |
| L9_3 | 1.665 | 108.333 | 0.999 | L9_3 | 1.226 | 144.625 | 1.000 |
| L13_1 | 1.615 | 105.250 | 0.999 | L13_1 | 1.423 | 108.200 | 1.000 |
| L13_2 | 1.423 | 59.273 | 1.000 | L13_2 | 1.420 | 110.333 | 1.000 |
| L13_3 | 1.433 | 90.667 | 0.999 | L13_3 | 1.371 | 116.000 | 1.000 |
| L17_1 | 1.073 | 90.111 | 0.999 | L17_1 | 1.461 | 63.500 | 1.000 |
| L17_2 | 1.205 | 103.000 | 0.999 | L17_2 | 1.240 | 58.250 | 1.000 |
| L17_3 | 1.081 | 70.000 | 1.000 | L17_3 | 1.211 | 57.500 | 1.000 |
| L21_1 | 0.800 | 73.000 | 1.000 | L21_1 | 0.544 | 54.000 | 1.000 |
| L21_2 | 1.079 | 83.714 | 1.000 | L21_2 | 0.706 | 54.000 | 1.000 |
| L21_3 | 0.805 | 71.273 | 1.000 | L21_3 | 0.860 | 51.000 | 1.000 |
| L24_1 | 1.329 | 97.000 | 0.999 | L24_1 | 1.377 | 100.583 | 1.000 |
| L24_2 | 0.911 | 96.000 | 0.999 | L24_2 | 0.986 | 70.000 | 1.000 |
| L24_3 | 0.840 | 75.000 | 1.000 | L24_3 | 1.014 | 88.750 | 1.000 |
| L27_1 | 0.937 | 86.769 | 0.999 | L27_1 | 0.764 | 71.500 | 1.000 |
| L27_2 | 0.866 | 92.813 | 0.999 | L27_2 | 1.047 | 72.500 | 1.000 |
| L27_3 | 1.033 | 90.545 | 0.999 | L27_3 | 1.009 | 84.429 | 1.000 |

**Table S2 Anosim of microbial community in samples from the same day with different fermentation depths.**

| Microbial community level | | Statistic | P-value |
| --- | --- | --- | --- |
| OTU | bacteria | 0.040 | 0.073 |
|  | fungi | -0.009 | 0.563 |
| Phylum | bacteria | 0.116 | 0.004 |
|  | fungi | -0.007 | 0.520 |
| Genus | bacteria | 0.055 | 0.025 |
|  | fungi | -0.027 | 0.900 |

# Supplementary Figure

**FIGURE S1** RDA/CCA analysis of pH/total acid content and bacterial community (A); The correlation between pH/total acid content and the top 10 bacterial genera (B); RDA/CCA analysis of pH/total acid content and fungal community (C); The correlation between pH/total acid content and the top 10 fungal genera (D). *: *P* < 0.05, **: *P* < 0.01, ***: *P* < 0.001.


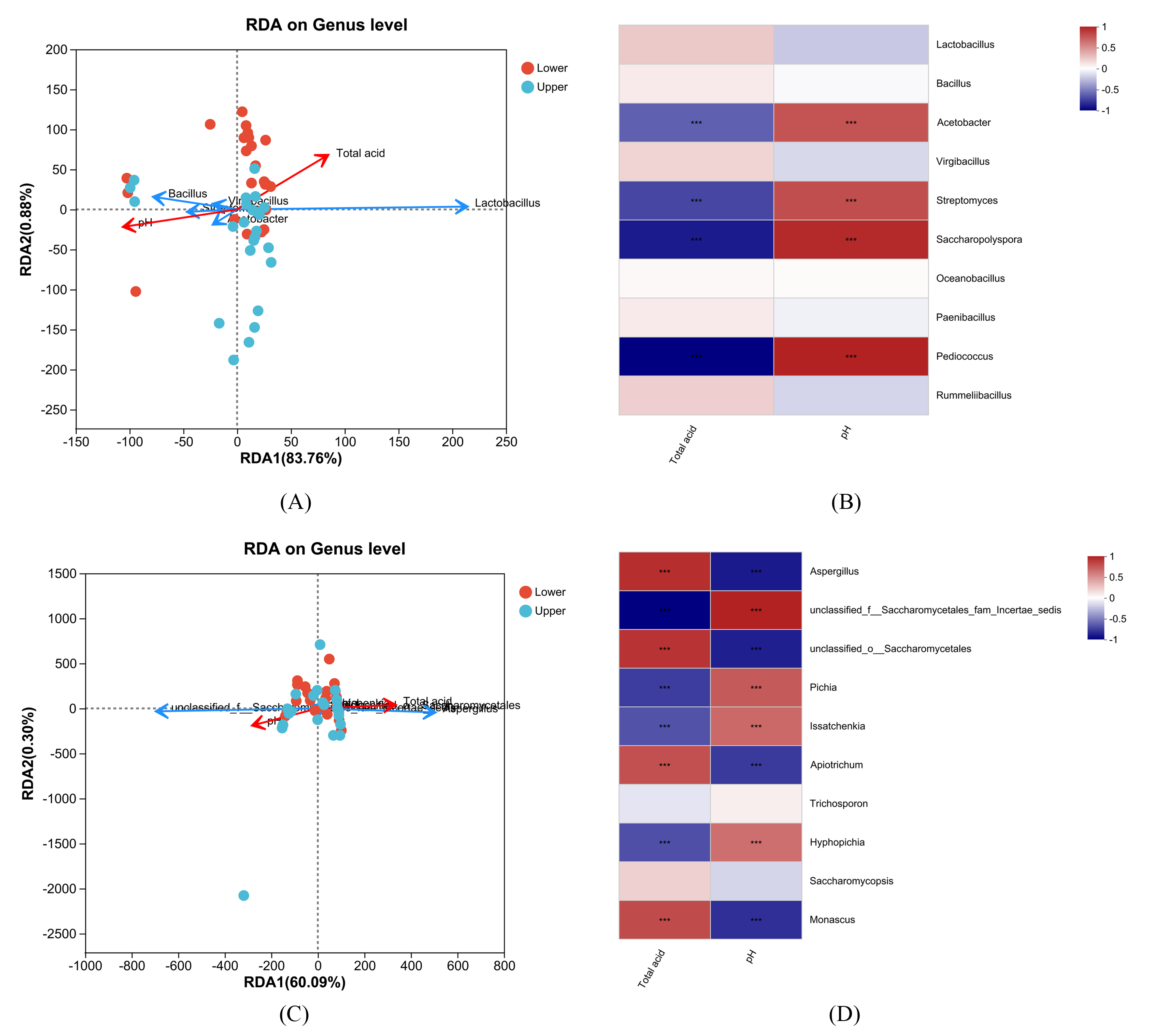

Supplement: Supplementary file 1 [file Data_Sheet_1.docx]
